# Round the Hospitals

**Published:** 1919-02-01

**Authors:** 


### ROUND THE HOSPITALS.

The College of Nursing has now moved to 7 Henrietta Street, Cavendish Square, which is immediately adjacent to its first home in Vere Street. Those who have had experience of the organisation of an office where many records are kept and made readily available will realise the difficulties under which the Secretariat have had to labour in their temporary offices. The new accommodation is contained in three floors of No. 7 Henrietta Street set apart for the work of the secretary, the organising secretary, and the registrar, in addition to a committee room and a waiting room on the ground floor. Telephones will connect the various departments, and all the rooms forming the various offices are bright, airy, and well arranged.

The first floor at 7 Henrietta Street constitutes the club rooms of the London Centre, which are comfortably furnished. There are plenty of easy chairs, sofas, and other comforts, and the walls are made attractive by good prints, some of which have been kindly lent, and here there is an opening for generous friends to make a few gifts to add to the general air of comfort and brightness which

**ROUND THE HOSPITALS**—(*continued*).

characterises this most inviting London Centre. Arrangements have been made that tea and coffee, cakes and sandwiches will be procurable between the hours of 10 and 6, and these club rooms are bound to become very popular with the members. The club was opened on January 23, when a pleasant reception was held before the Registration meeting. A large number of representative members of the nursing profession attended and seemed thoroughly delighted with their surroundings. At the conclusion of the meeting at the Royal Society of Medicine, Miss Montgomery, matron of the Middlesex Hospital and chairman of the London Local Centre, gave all College members a cordial invitation to join the Centre and thus gain the advantage of the use of the club rooms. The subscription is 5s. a year, which should be sent to the Honorary Secretary, Miss Biggar, at St. Thomas's Hospital, S.E. It is hoped that members will make full and constant use of the club rooms, to which they may bring friends, even though these friends are not members of the College.

At a meeting of the board of management on Tuesday, Miss Amelia Smith, assistant matron of King Edward's Hospital, Cardiff, was appointed matron of Northampton General Hospital. There were fifty-three candidates. Miss Smith is thirty-four years of age, and has been engaged in hospital work since she was twenty. Commencing her training at Cambridge, she filled with great ability successive appointments at Wakefield, Bury, Essex County Hospital, Birkenhead, and Dundee, before going to Cardiff in March 1917. Miss Smith enters on her new duties at the beginning of March.

General Sir E. H. Allenby, G.C.B., G.C.M.G., Commander-in-Chief, Expeditionary Force, has forwarded to the Secretary of State for War, under date of October 23, 1918, a list of officers, nurses, other ranks, and civilians whom he considers worthy of mention for their services during the period from March 16, 1918, to September 18, 1918. This list includes the following:—*Queen Alexandra's Imperial Military Nursing Service*: Sister (Acting Matron) Miss K. F. G. Skinner, R.R.C. *The Reserve*: Superintendent Nurses Miss F. M. Macdonald and Miss M. Murphy. *Territorial Force Nursing Service*: Sister A. Brown. *Voluntary Aid Detachment*: Nurse Miss N. G. Evans. *Australian Imperial Force, Red Cross Society*: Mrs. L. T. Granville. *New Zealand Force, Army Nursing Service*: Sister K. Booth.

The demand for nurses continues to be acute. A large number of important institutional posts are vacant and more school-nurses, child welfare superintendents and public health workers are needed than can be supplied. It is probable that the gradual tranquillisation of the world will lead to a very large extension of nursing work in countries where at present the facilities for training are very scanty and women of culture with open minds and adventurous dispositions will be needed as pioneers. There is a call to relax the rigidity of the nurse training-schools, at any rate in the fourth year, so that the right kind of women may be prepared in the right kind of way for public posts at home and abroad.

A plucky attempt is being made to rescue domestic service from the ill-odour into which it has fallen among the girls who used to adopt it as their career. Lady Londonderry, President of the Women's Legion, and Dame Burleigh Leach, Controller-in-Chief of Queen Mary's Army Auxiliary Corps, have evolved a scheme under which "Legionaries" will take domestic duties, standardised by conditions previously laid down and at a stated rate of pay. The principal point consists in leave regulations. There are to be two hours' leave daily, within working hours, irrespective of meal times. This is by no means an unreasonable stipulation, and it is one frequently accorded in institutions where the maids are really considered. Nor are the rates of pay unreasonable, ranging as they do from £50 for a housekeeper, £28-£45 for a cook, and £18-30 for a housemaid according to status. It is hoped to extend the League to embrace a section for club and hotel servants, and we trust the promoters of the scheme will not ignore the pressing needs of hospitals and nursing institutions.

We observe a great many civil speeches from Guardians and other managers of institutions on the stupendous exertions undertaken by certain members of the nursing staff during the influenza epidemic when their colleagues were laid low. What would their gratitude amount to if expressed in money at so much an hour overtime? It would be interesting to know. We are disposed to think that many abuses in connection with the surrender of off-duty time at the call of necessity would disappear if nurses had the same privileges as other workers and were paid extra when called on to work beyond the stipulated hours. In some instances an extra week's holiday has been allowed in lieu of or in addition to compliments, but we should like to see a money payment as well, the one as a bare matter of justice, the other as an act of grace and gratitude.

Somewhat contrary to expectation the inquiries received at Devonshire House relating to the V.A.D. scholarship scheme show that in the choice of future work the members of Voluntary Aid Detachments lean most to nursing. A good number of candidates desire to take up public health work, and some desire to qualify in pharmacy. The work of demobilisation is proceeding but slowly, for though the American and New Zealand hospitals are being closed with most of the smaller auxiliary hospitals, many military hospitals are still short-handed and are asking for members. Lady Ampthill has issued a preliminary list of careers for which V.A.D. workers would seem specially suitable, with the approximate period of training and the probable salary to be gained when fully trained. Every effort will be made to keep these patriotic women from drifting into blind-alley occupations.

**ROUND THE HOSPITALS—(*continued*).**

The American Red Cross has done a wise and generous deed in devoting part of the proceeds of the Children's Jewel Fund to the conversion of a public-house in Bethnal Green to a Child-Welfare Centre. There is to be an ante-natal clinic later on in connection with the institution, which was opened on January 24 by the Duchess of Marlborough, as Honorary Treasurer of the Children's Jewel Fund. Two women doctors, assisted by fully-qualified nurses, are in charge of the centre, which is situated in a side street in a densely-populated and poor quarter, close to the doors of those for whose benefit it is intended. Much of the success of such enterprises depends on securing a good matron to throw her whole interest into the work. Managers cannot too clearly understand that to begin by paying an adequate salary to a capable matron of heart and understanding is the only economical way of running a Child-Welfare Centre.

---

Nearly 13,000 graduate nurses, nurses' aides, practical nurses, and volunteers were enlisted by the Red Cross in America to stem the influenza epidemic, and considering the important part which the "aides" took in the campaign the utility of short courses of training even in peace-time is fully vindicated. The death-rate in the United States from influenza reached its height during the two weeks ended October 26, for which period 40,782 deaths were recorded. Miss Hilliard, President of the New York City League for Nursing Education, writes warmly in the January number of the *Trained Nurse* on the self-forgetfulness and self-sacrifice displayed by the student nurses or probationers in the New York hospitals during the epidemic. She writes, "There was no question here of compensation or reward, as student nurses always give their services freely to the hospitals in return for their training." It is sad to learn that at least twenty-seven nurses, students, and graduates died during the epidemic in New York alone. We are not aware that any computation has been made of the number of English nurses in London who made the supreme sacrifice in the course of their battle with the infection. It is a matter of some moment to ascertain this fact.

---

The Young Women's Christian Association has conferred an admirable service on educated women in carrying through the first part of their scheme for emergency hostels intended for their benefit. Queen Mary has taken considerable interest in the idea as originally sketched. She has given £100 towards the funds and has allowed the hostels to be named after her. They are designed to afford accommodation for women of small means desirous of spending a few days in town on business. The charge for bed and breakfast is 3s. 6d. to 5s., and it is anticipated the hostels will be in great request. The first one has been opened at 37 Brunswick Square and others are in course of preparation in Mecklenburg Square and in Lexham Gardens. It is hoped to have a widespread system of Queen Mary Emergency Hostels connected by telephone in order that women workers may be spared the trials of hunting for rooms and finding none on their arrival in town.

---

The inquest involving cocaine which has ended in a verdict of manslaughter against one of the principal witnesses is chiefly interesting from the vista it opens up of the dangerous influences which threaten the women working for public amusement. There is something peculiarly revolting in the circumstances which condemn the young, gay, pretty girls who adopt the stage as their career to association with degenerates of the lowest type. Generation after generation of these popular favourites, gifted with unusual graces, fitted for happy domestic surroundings, are simply broken on the wheel by the outrageous manner of life into which they are dragged by evil-minded men and women who study to use them for their purposes. In no direction are English people more culpably helpless than in permitting their amusements to be the means of the wreck of young lives. Compared with the process under which this girl of twenty-three was slowly done to death in mind and body a Spanish bull-fight is humanity itself.

---

An inquest was held on the body of Nursing Sister Lilian Alice Houseley by the York Coroner on January 22, from which it transpired that the deceased, who suffered from heart attacks, had been overtaken by syncope while in the bath and died from drowning. She was on the eve of her marriage, and had lately left the Auxiliary Military Hospital, Caerphilly. Her sister gave evidence to the effect that Sister Houseley had mentioned straining her heart through overwork two years ago. A prolonged rest at that time might have preserved a life valuable to the community.

---

The evacuation by the War Office of the civil institutions appropriated during the war for the care of naval and military patients is proceeding with aggravating unevenness and delay. At York the Poor-Law authorities have got back their infirmary with a handsome acknowledgment of the Guardians' kindness in placing it for so long at the disposal of the War Office. But at Oxford they are in sore straits for want of their infirmary, which is still in the occupation of the military. All the outlying infirmaries to which Oxford patients must now be sent are full to overflowing, and the Provost of Oriel, presiding at a meeting of the Oxford Guardians, stated that there was an alarming number of cases awaiting treatment in the town. Much of the delay in getting back to pre-war conditions appears to be avoidable. It presses very hardly in many instances on the nurses, who are unable to make plans or accept posts until the date when their institution will be demobilised is fixed.

---

At an animated meeting held in Hastings on January 21, it was resolved that the war memorial

ROUND THE HOSPITALS—(*continued*).

for the borough should take the form of a public monument to be erected on a central site, together with financial assistance to the East Sussex and the Buchanan Hospitals, in the proportion of two-thirds to the former institution and one-third to the latter. The foundations in ferro-concrete of the new East Sussex Hospital have been already laid, and it is estimated that the work of completing the building and equipping it could be completed within two years if put in hand at once. The cost is estimated at £90,000, of which some £50,000 have still to be raised. There was evidently a strong feeling at the meeting in favour of making hospital extension the main feature of the memorial, and one lady present, speaking as "a mother who knew what it was to lose," maintained that those who had fallen would like a living monument "associated with nurses." There can be no doubt that if the case for the hospitals were adequately brought before the public very much larger support could be elicited from this wealthy neighbourhood than has hitherto been forthcoming. The result of one aptly worded letter in the local press ventilating the need for a cottage hospital at Rye was that cheques to the amount of £3,600 were received within a few days from Winchelsea alone.

---

CERTAIN special homes have kept going with extreme difficulty during the past year, and among these is St. Mary's Home, Buxted, for children from four to ten years of age who have fallen victims to vice. The plain and terrible fact of the increasing number of criminal assaults against young children, some of whom are victims in their own families, has at length forced itself on the knowledge of the public. Among the preventive measures established none to our knowledge has borne richer fruit in the restoration to happy childhood of the unhappy sufferers than St. Mary's Home. These children cannot be dealt with in ordinary schools. They need most careful and special training—above all, they need love. The Home is entirely unendowed and receives thirty children. The payment received for each child hardly now covers the expenses for board alone, and it is highly creditable to the sister-in-charge that the past year has closed without debt. That the Home should be relieved from the incessant burden of anxiety about ways and means and undergo extension must be the wish of all who know its trials and its amazing successes in the task of helping to wipe out a national disgrace. Thirty-three applications had perforce to be refused last year.

---

IN the Plymouth district, owing to the serious shortage of staff nurses, nursing has been carried on lately under "almost impossible conditions." The Three Towns Nursing Association and Training School for Nurses at its recent annual meeting reported, nevertheless, a year of substantial progress. Nearly 75,000 visits—a record number—were paid by the staff, and the training centre sends out nurses for nearly the whole of Cornwall. A new Maternity Home is in course of construction, and an Infants Welfare Centre is fully established and doing valuable work. The managers are experiencing a good deal of difficulty in securing funds adequate for their many-sided work, but they have enlisted the services of some very enthusiastic workers on their behalf, and no one doubts that the deficit shown in the balance-sheet will speedily be wiped out.

---

BEXHILL, the youngest of the Sussex boroughs, is moving with the times. It has just decided upon the establishment of a nursing association (to be affiliated to the Queen Victoria Jubilee Institute for Nurses and the East Sussex County Nursing Association), with the object of undertaking all the nursing and health work contemplated by the Maternity and Child-Welfare Act, 1918, together with the provision of midwives and such public health nursing as they might think proper to undertake with the approval of the local authority. That the meeting at which this decision was arrived at was held under civic auspices, that the Mayor presided, and that doctors and clergy united in approval are an earnest of the desire of Bexhill to rid itself of the existing unsatisfactory system by which cases are often visited by agents of two, three, or four different organisations. These various agencies have all done excellent work individually, but co-ordination means greater efficiency than can be attained when the same work is carried out by a multiplication of small bodies.

---

THE Food Controller announces that there is no immediate prospect of any reduction in food prices. This means, we fear, that institutions desirous of adding to the number of their nurses will hesitate a little longer before shouldering the heavy burden involved in the cost of board. Even under normal circumstances something like scarcity often prevails during the two or three months before us when the vegetable supplies are beginning to run out, when stormy weather lessens the fish supply, and poultry is almost unattainable. The situation, however, is being relieved by the gradual relaxation of the rationing orders. More sugar means more puddings, and the escape of bacon and "offal" from the iron grip has eased the housekeeper's task if it has tended to increase the weekly bills. With the arrival of summer we may begin to realise that the worst is over.

---

☞ IT is our invariable practice to pay for all items of news which we may publish. The minimum payment is 5s. Paragraphs of about twenty lines in length—that is to say, of 150 words or less—are preferred. Contributions should be addressed to The Editor, THE HOSPITAL, 28 and 29 Southampton Street, Strand, London, W.C. 2, and, to be in time for the current week's issue, should reach him by the first post on Mondays.

